# Retroflexed endoscopic submucosal dissection of a lesion invading the pyloric ring, using a newly developed thin endoscope

**DOI:** 10.1055/a-2248-0688

**Published:** 2024-02-15

**Authors:** Satoki Shichijo, Daiki Kitagawa, Yuya Asada, Shunsuke Yoshii, Noriya Uedo, Ryu Ishihara, Tomoki Michida

**Affiliations:** 1 53312 Gastrointestinal Oncology, Osaka International Cancer Institute, Osaka, Japan; 2 Gastroenterology, Osaka Metropolitan University Graduate School of Medicine, Osaka, Japan

Endoscopic resection of a pyloric lesion is challenging because of the limited maneuverability of endoscopes. Here, we report a case of a depressed pyloric lesion treated with endoscopic resection using a newly developed thin endoscope.

A 74-year-old woman who had previously undergone *Helicobacter pylori* eradication was found to have a pyloric lesion on follow-up endoscopy. Biopsy suggested that the lesion was an adenocarcinoma, and the patient was referred to our institute for further management.

The lesion was located in the posterior region of the pylorus, invading the pyloric ring ( Fig. 1 ). Another lesion was identified on the anterior wall of the pylorus. Considering the absence of signs of invasion, we performed endoscopic resection of the lesion ( Video 1 ).

**Fig. 1:**
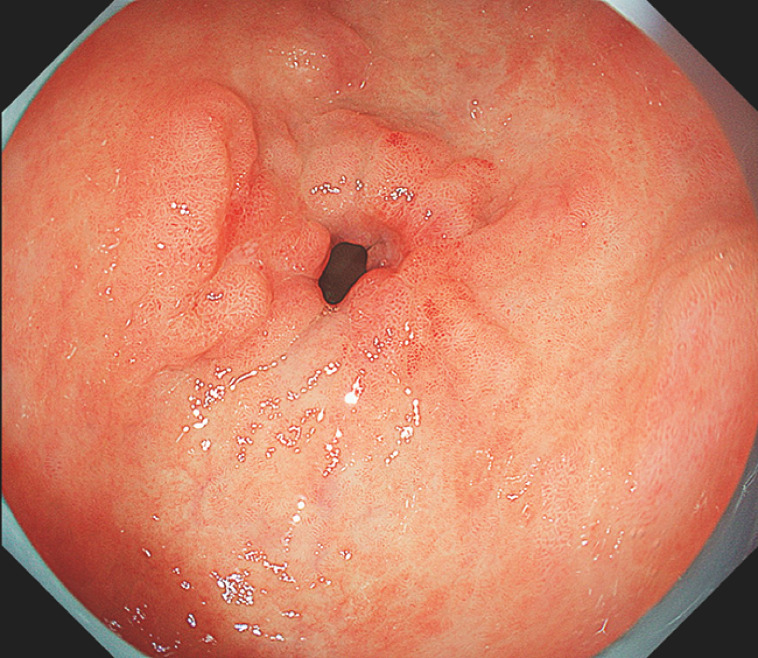
Endoscopic view of a lesion located in the posterior pyloric region, invading the pyloric ring, and another lesion located on the anterior pyloric wall.

Retroflexed endoscopic submucosal dissection of a lesion invading the pyloric ring, using a newly developed thin endoscope.Video 1

First, we made markings on the oral side using magnifying endoscopy (GIF-XZ1200; Olympus Co. Ltd., Tokyo, Japan). The tumor had invaded the posterior wall of the duodenal bulb, and the margin could not be identified in the forward view ( Fig. 2 ). Retroflexed observation was impossible because of the narrow space of the bulb. Therefore, we used a newly developed thin endoscope (EG-840TP; Fujifilm, Tokyo, Japan), with a width of 7.9 mm, large working channel of 3.2 mm, and wide angles (up, 210°; down, 160°) 1 . We made circumferential markings in the retroflexed view and performed endoscopic submucosal dissection (ESD) from the anal side ( Fig. 3 ). Subsequently, ESD was performed from the oral side using an endoscope with an attached hood (DH-083ST; Fujifilm). Using the pulley traction method ( Fig. 4 ) 2 3 4 5 , we successfully achieved en bloc resection of the lesion ( Fig. 5 ).

**Fig. 2:**
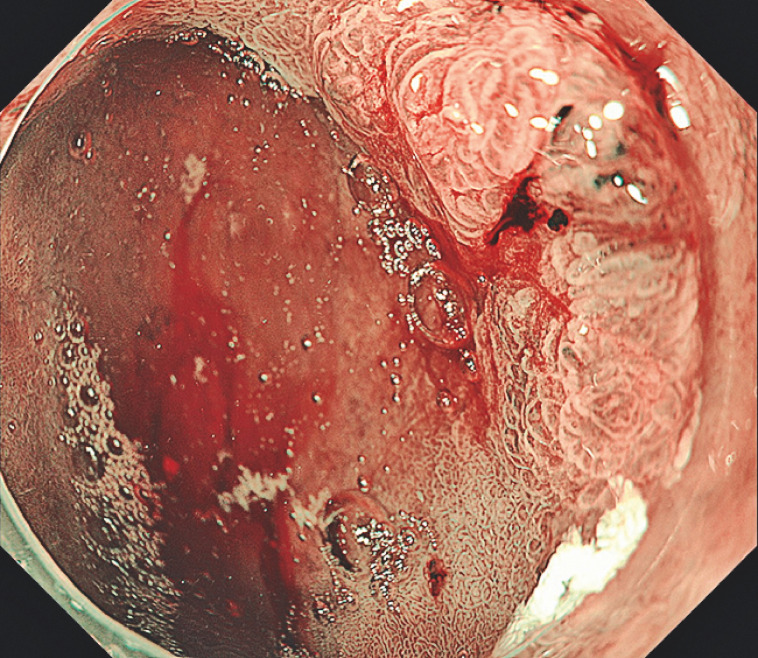
Endoscopic view of a tumor invading the posterior wall of the duodenal bulb.

**Fig. 3:**
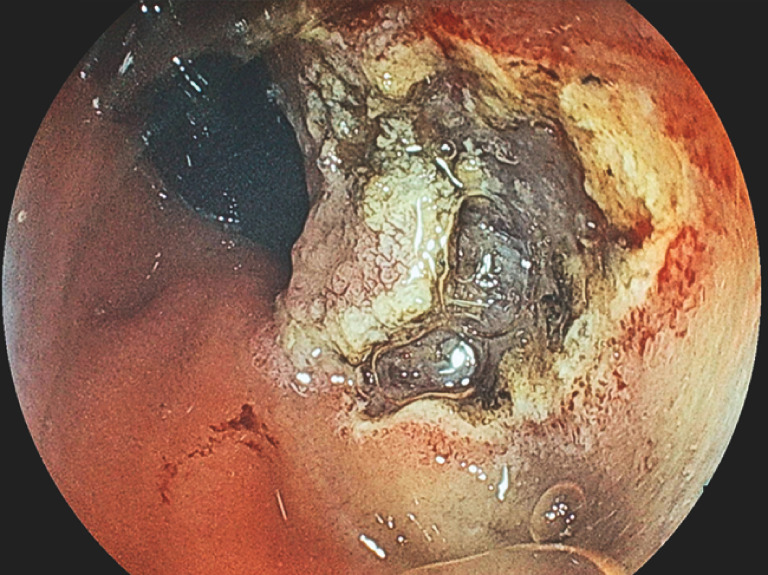
Endoscopic view of endoscopic submucosal dissection being performed from the anal side.

**Fig. 4:**
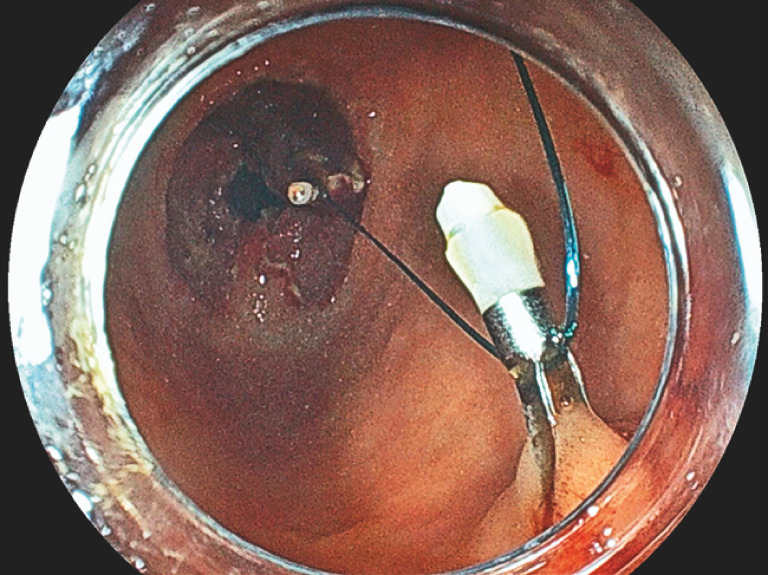
Endoscopic view demonstrating the pulley traction method.

**Fig. 5:**
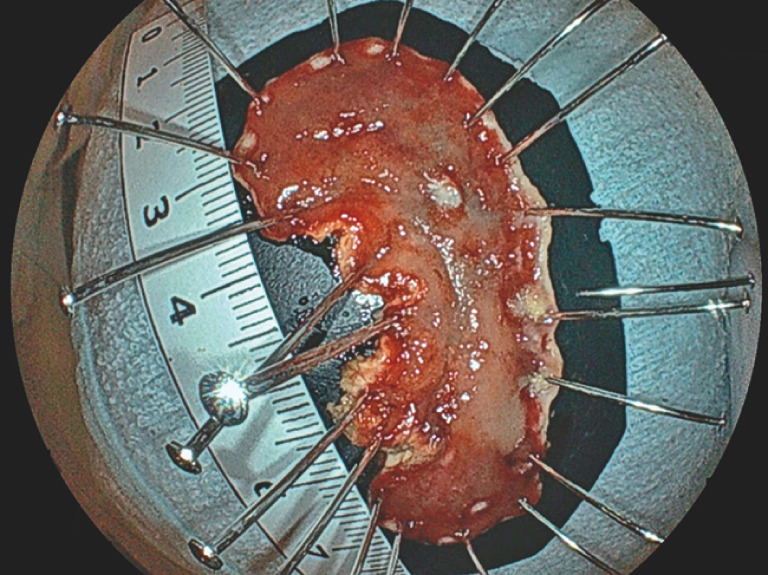
Endoscopic view showing the resected specimen after en bloc resection of the lesion.

The final pathological diagnosis was 0–IIc, 29 × 15-mm, well-differentiated tubular adenocarcinoma, pT1a, pUL1, ly0, v0, pHM0, pVM0, with the two lesions considered continuous.

Endoscopy_UCTN_Code_TTT_1AO_2AG

## Notes

Endoscopy E-Videos https://eref.thieme.de/e-videos
*E-Videos* is an open access online section of the journal *Endoscopy* , reporting on interesting cases and new techniques in gastroenterological endoscopy. All papers include a high-quality video and are published with a Creative Commons CC-BY license. Endoscopy E-Videos qualify for HINARI discounts and waivers and eligibility is automatically checked during the submission process. We grant 100% waivers to articles whose corresponding authors are based in Group A countries and 50% waivers to those who are based in Group B countries as classified by Research4Life (see: https://www.research4life.org/access/eligibility/ ). This section has its own submission website at https://mc.manuscriptcentral.com/e-videos .
